# Inhibition of acid sphingomyelinase by ambroxol prevents SARS-CoV-2 entry into epithelial cells

**DOI:** 10.1016/j.jbc.2021.100701

**Published:** 2021-04-23

**Authors:** Alexander Carpinteiro, Barbara Gripp, Markus Hoffmann, Stefan Pöhlmann, Nicolas Hoertel, Michael J. Edwards, Markus Kamler, Johannes Kornhuber, Katrin Anne Becker, Erich Gulbins

**Affiliations:** 1 Institute of Molecular Biology, University of Duisburg-Essen, Essen, Germany; 2 Department of Hematology, University Hospital Essen, University of Duisburg-Essen, Essen, Germany; 3 Zentrum für Seelische Gesundheit des Kindes- und Jugendalters, Sana-Klinikum Remscheid GmbH, Remscheid, Germany; 4 Infection Biology Unit, German Primate Center – Leibniz Institute for Primate Research, Göttingen, Germany; 5 Faculty of Biology and Psychology, University of Göttingen, Göttingen, Germany; 6 AP-HP.Centre-Université de Paris, Hôpital Corentin-Celton, Département de Psychiatrie, Issy-les-Moulineaux, and Université de Paris, INSERM, Institut de Psychiatrie et Neurosciences de Paris, UMR_S1266, and Faculté de Santé, UFR de Médecine, Paris, France; 7 Department of Surgery, Medical School, University of Cincinnati, Cincinnati, Ohio, USA; 8 Department of Thoracic and Cardiovascular Surgery, Division of Thoracic Transplantation, University Hospital Essen, Essen, Germany; 9 Department of Psychiatry and Psychotherapy, University Hospital of Erlangen, Friedrich-Alexander University Erlangen-Nürnberg (FAU), Erlangen, Germany

**Keywords:** ambroxol, SARS-CoV-2, acid sphingomyelinase, ceramide, infection, human nasal epithelial cells, ACE2, angiotensin-converting enzyme 2, ATCC, American Type Culture Collection, COVID-19, coronavirus disease 2019, DMEM, Dulbecco's modified Eagle's medium, DTPA, diethylenetriaminepentaacetic acid, eGFP, enhanced GFP, FCS, fetal calf serum, FIASMAs, functional inhibitors of the acid sphingomyelinase, H/S, Hepes/saline, MEM, minimum essential medium, NP-40, Nonidet P40, OGP, *n*-octyl-d-glucopyranoside, PFA, paraformaldehyde, pp-VSV-SARS-CoV-2, vesicular stomatitis virus pseudoviral particles presenting SARS-CoV-2 spike protein on their surface, SARS-CoV-2, severe acute respiratory syndrome coronavirus 2, TMPRSS2, transmembrane serine protease 2

## Abstract

The acid sphingomyelinase/ceramide system has been shown to be important for cellular infection with at least some viruses, for instance, rhinovirus or severe acute respiratory syndrome coronavirus 2 (SARS-CoV-2). Functional inhibition of the acid sphingomyelinase using tricyclic antidepressants prevented infection of epithelial cells, for instance with SARS-CoV-2. The structure of ambroxol, that is, *trans*-4-[(2,4-dibromanilin-6-yl)-methyamino]-cyclohexanol, a mucolytic drug applied by inhalation, suggests that the drug might inhibit the acid sphingomyelinase and thereby infection with SARS-CoV-2. To test this, we used vesicular stomatitis virus pseudoviral particles presenting SARS-CoV-2 spike protein on their surface (pp-VSV-SARS-CoV-2 spike), a *bona fide* system for mimicking SARS-CoV-2 entry into cells. Viral uptake and formation of ceramide localization were determined by fluorescence microscopy, activity of the acid sphingomyelinase by consumption of [^14^C]sphingomyelin and ceramide was quantified by a kinase method. We found that entry of pp-VSV-SARS-CoV-2 spike required activation of acid sphingomyelinase and release of ceramide, events that were all prevented by pretreatment with ambroxol. We also obtained nasal epithelial cells from human volunteers prior to and after inhalation of ambroxol. Inhalation of ambroxol reduced acid sphingomyelinase activity in nasal epithelial cells and prevented pp-VSV-SARS-CoV-2 spike-induced acid sphingomyelinase activation, ceramide release, and entry of pp-VSV-SARS-CoV-2 spike *ex vivo*. The addition of purified acid sphingomyelinase or C16 ceramide restored entry of pp-VSV-SARS-CoV-2 spike into ambroxol-treated epithelial cells. We propose that ambroxol might be suitable for clinical studies to prevent coronavirus disease 2019.

Infections with severe acute respiratory syndrome coronavirus 2 (SARS-CoV-2) is responsible for the coronavirus disease 2019 (COVID-19) pandemic, a massive global health problem. Many infected patients exhibit mild symptoms, but a substantial number of patients, in particular older patients and those with additional risk factors, develop severe disease (1). Severe COVID-19 requires intensive care and ventilator treatment and is associated with a high mortality rate (1). Age, high blood pressure, or overweight are risk factors for severe COVID-19, but even healthy and young individuals develop severe disease (2). It is therefore of outstanding interest to develop robust prophylaxis and treatment options.

The SARS-CoV-2 spike protein is incorporated into the viral envelope and mediates viral entry into target cells. For this, the surface unit S1 of SARS-CoV-2 spike binds to the cellular receptor angiotensin-converting enzyme 2 (ACE2) (3, 4, 5), and the S2 subunit fuses the viral membrane with a target cell membrane. The structural requirements of the interaction of the spike protein with ACE2 are well characterized (3, 4, 5). However, the role of membrane lipids for viral entry requires definition.

Sphingolipids are not only structural components of cellular membranes and determine their biophysical membrane properties, but they are also involved in cellular signaling transduction, regulation of proliferation and differentiation, apoptosis, membrane trafficking, and the organization of proteins within membranes (6, 7, 8, 9, 10). Here, we focused on the role acid sphingomyelinase (Enzyme Commission no.: 3.1.4.12, sphingomyelin phosphodiesterase), a lysosomal enzyme that converts sphingomyelin into ceramide, plays in cellular infection with SARS-CoV-2. The enzyme is present in lysosomes and acidic domains of the cell membrane upon fusion of secretory lysosomes with the plasma membrane (10, 11). The activity of acid sphingomyelinase results in the release of ceramide from sphingomyelin and, if ceramide is released on the cell surface, in the formation of ceramide-enriched membrane domains in the outer leaflet of the cell membrane (9, 10, 11, 12, 13). The very hydrophobic ceramide molecules associate with each other to spontaneously form distinct membrane domains. These ceramide-enriched membrane domains fuse into large highly hydrophobic, tightly packed, and gel-like ceramide-enriched membrane platforms (10, 11). These platforms serve to cluster receptor molecules and to organize, trap, and concentrate specific signaling molecules (10, 11, 12, 13).

We have previously shown an important role of the acid sphingomyelinase/ceramide system for SARS-CoV-2 infections (14). We demonstrated that infection of cultured epithelial cells or freshly isolated human nasal epithelial cells with SARS-CoV-2 or vesicular stomatitis virus (VSV) pseudoviral (pp-VSV-SARS-CoV-2) spike particles, respectively, resulted in activation of the acid sphingomyelinase and release of ceramide (14). Genetic or pharmacological inhibition of the acid sphingomyelinase as well as consumption or neutralization of ceramide on the cell surface prevented infection of epithelial cells with SARS-CoV-2 (14).

Several drugs functionally inhibit the acid sphingomyelinase (15, 16, 17, 18, 19, 20, 21). Structural requirements for inhibition are a lipophilic organic ring that integrates into lysosomal membranes, a short spacer, and a charged tertiary amine group that displaces the acid sphingomyelinase from the lysosomal membranes, thereby releasing the enzyme into the lysosomal lumen and causing its partial degradation. Many antidepressants are functional inhibitors of the acid sphingomyelinase (FIASMAs) (15, 16, 17, 18, 19, 20, 21). We have proposed the acronym FIASMA (Functional Inhibitor of Acid SphingoMyelinAse) for a compound from this large group of drugs with these properties (21).

Here, we tested whether ambroxol, that is, *trans*-4-[(2,4-dibromanilin-6-yl)-methyamino]-cyclohexanol, a drug that fits with the aforementioned structural requirements, inhibits acid sphingomyelinase and can thus be repurposed to inhibit SARS-CoV-2 infection. Ambroxol contains a lipophilic organic ring system that is connected to a tertiary amine *via* a short spacer and, therefore, is a potential functional inhibitor of the acid sphingomyelinase. Ambroxol is a mucolytic drug that is used for the treatment of diseases of the upper and lower respiratory tract. It has almost no side effects.

As an infection system, we used replication-deficient VSV pseudoviral particles (pp-VSV) presenting SARS-CoV-2 spike protein on their surface, abbreviated pp-VSV-SARS-CoV-2 spike (22). Several previous studies showed that these particles accurately reflect key aspects of the entry of coronavirus into host cells (14, 22).

We demonstrate that entry of pp-VSV-SARS-CoV-2 spike into cultured epithelial cells or freshly isolated nasal epithelial cells results in activation of the acid sphingomyelinase and a release of ceramide. These events were blocked by pretreatment with low doses of ambroxol. In accordance, ambroxol prevented cellular entry of pp-VSV-SARS-CoV-2 spike. More importantly, we obtained nasal epithelial cells from volunteers prior and after inhalation with ambroxol and infected the cells with pp-VSV-SARS-CoV-2 spike. These *in vivo*/*ex vivo* experiments demonstrated that inhalation of ambroxol is sufficient to reduce acid sphingomyelinase activity in nasal epithelial cells *in vivo* and to prevent infection with pp-VSV-SARS-CoV-2 spike *ex vivo*. Addition of purified acid sphingomyelinase or C16 ceramide restored infection of ambroxol-treated nasal epithelial cells with pp-VSV-SARS-CoV-2 spike.

## Results

**Figure 1:**
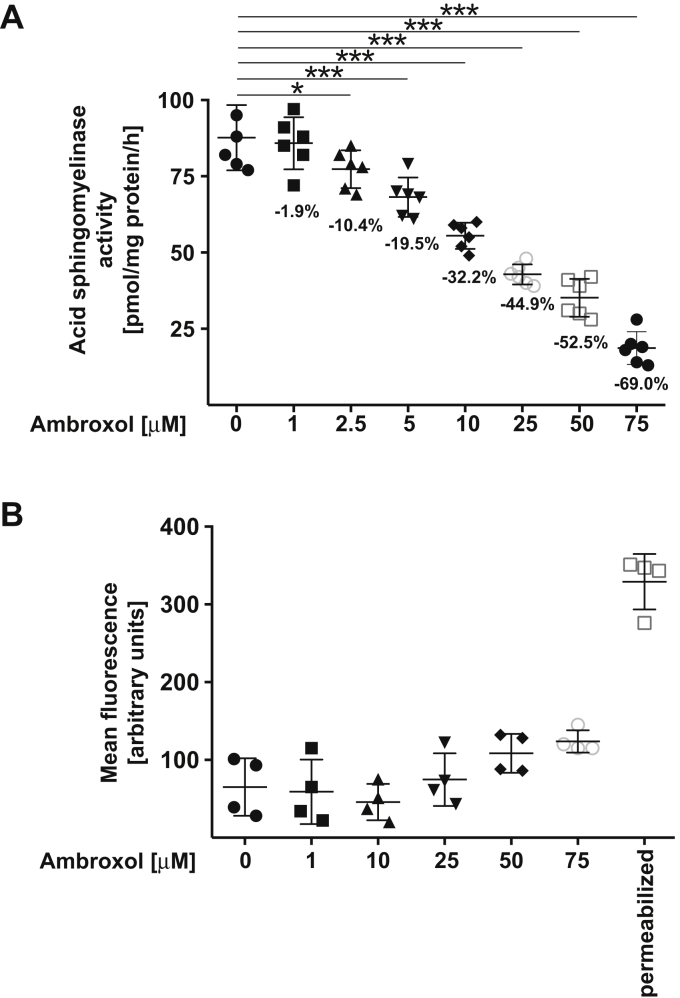
**Ambroxol reduces the activity of the acid sphingomyelinase.***A*, Vero-E6 cells were incubated with 1, 2.5, 5, 10, 25, 50, or 75 μM ambroxol or with solvent (0) for 60 min. Cells were lysed in 250 mM sodium acetate (pH 5.0) and 0.2% NP-40, and acid sphingomyelinase activity was determined by measuring the consumption of added [^14^C]sphingomyelin. The percent decrease of acid sphingomyelinase activity is given for easier quantification. Shown are the means ± SD of the acid sphingomyelinase activities from six independent experiments. ∗*p* < 0.05, ∗∗∗*p* < 0.001, ANOVA followed by post hoc Student's *t* tests. *B*, ambroxol did not exhibit cytotoxicity after treatment for 24 h. Cells were exposed for 24 h to 1, 10, 25, 50, or 75 μM ambroxol, and toxicity was measured by FITC–annexin V staining. FITC–annexin V staining was analyzed by flow cytometry. Permeabilized cells served as positive controls for the FITC–annexin V staining. Shown are means ± SD of the fluorescence (in arbitrary units) in the flow cytometry studies (n = 4); ANOVA, followed by post hoc Student's *t* tests. Results are presented in arbitrary units (a.u.). NP-40, Nonidet P40.

To test whether ambroxol inhibits the acid sphingomyelinase, we incubated Vero-E6 epithelial cells with increasing doses of ambroxol and determined the activity of the acid sphingomyelinase in cell lysates. Ambroxol induced a dose-dependent reduction of the activity of the acid sphingomyelinase in Vero-E6 cells (Fig. 1*A*). Ambroxol did not show toxicity until 50 μM concentration, whereas higher concentrations such as 75 μM started to show some toxicity as evidenced by flow cytometry studies of untreated and ambroxol-treated cells stained with FITC–annexin V (Roche; Fig. 1*B*).

**Figure 2:**
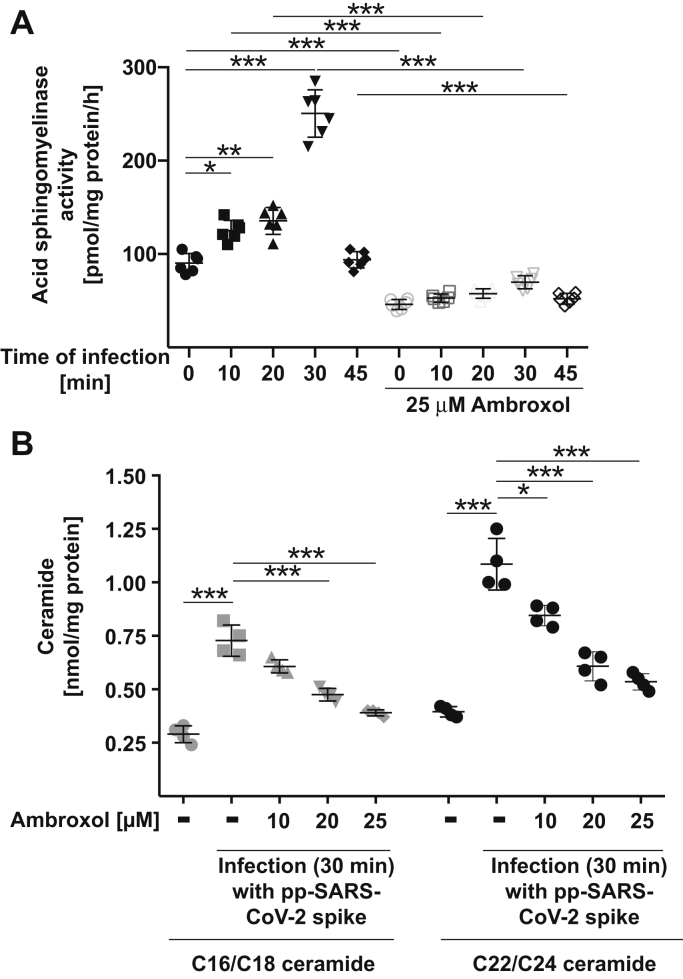
**Ambroxol prevents activation of the acid sphingomyelinase and release of ceramide upon infection with pp-VSV-SARS-CoV-2 spike.***A*, Vero-E6 cells were preincubated with 25 μM ambroxol for 60 min or were left untreated. Cells were infected with pp-VSV-SARS-CoV-2 spike for the indicated times or were left uninfected (0). Cells were lysed in 250 mM sodium acetate (pH 5.0) and 0.2% NP-40, and acid sphingomyelinase activity was determined by measuring the consumption of added [^14^C]sphingomyelin. Displayed are the means ± SD of the activity of the acid sphingomyelinase from six independent experiments. ∗*p* < 0.05, ∗∗*p* < 0.01, ∗∗∗*p* < 0.001, ANOVA followed by post hoc Student's *t* tests. *Closed symbols* give the time course without ambroxol, and *open symbols* give the time course with 25 μM ambroxol. *B*, cells were treated with 10, 20, or 25 μM ambroxol for 1 h and infected with pp-SARS-CoV-2 spike for 30 min or left untreated or uninfected, washed, and organically extracted to quantify C16/C18 ceramide and C22/C24 ceramide levels by using the ceramide kinase method. Displayed are the means ± SD of the ceramide concentrations from each six independent experiments. ∗*p* < 0.05, ∗∗∗*p* < 0.001, ANOVA, followed by post hoc Student's *t* tests, as indicated or compared with the corresponding value without inhibitor. *Gray symbols* represent 16/C18 ceramides, and *black symbols* represent C22/24 ceramides. NP-40, Nonidet P40; pp-VSV-SARS-CoV-2, vesicular stomatitis virus pseudoviral particles presenting SARS-CoV-2 spike protein on their surface.

Next, we investigated whether pp-VSV-SARS-CoV-2 spike induces an activation of the acid sphingomyelinase in Vero-E6 cells and whether this increase is prevented by pretreatment of the cells with ambroxol. Infection of the cells with pp-VSV-SARS-CoV-2 spike resulted in a rapid activation of the acid sphingomyelinase that peaked 30 min after infection (Fig. 2*A*), consistent with previously reported data (14). The increase of acid sphingomyelinase activity upon infection with pp-VSV-SARS-CoV-2 spike was prevented by preincubation with ambroxol (Fig. 2*A*). Ambroxol had no effect on acid ceramidase activity (not shown).

We have previously shown that incubation of epithelial cells with pp-VSV-SARS-CoV-2 spike triggers a release of ceramide that is essential for cellular infection with the virus (14). Here, we confirm these data and show that infection of Vero-E6 cells with pp-VSV-SARS-CoV-2 spike results in a release of C16/C18 ceramide as well as C22/C24 ceramide (Fig. 2*B*). The release of ceramide upon infection is prevented by pretreatment with ambroxol (Fig. 2*B*). We observed a complete inhibition of infection-induced ceramide formation at 25 μM, but already concentrations of 15 to 20 μM ambroxol reduced or almost abrogated ceramide formation upon infection (Fig. 2*B*). Ambroxol did not change basal ceramide concentrations of the cells.

**Figure 3:**
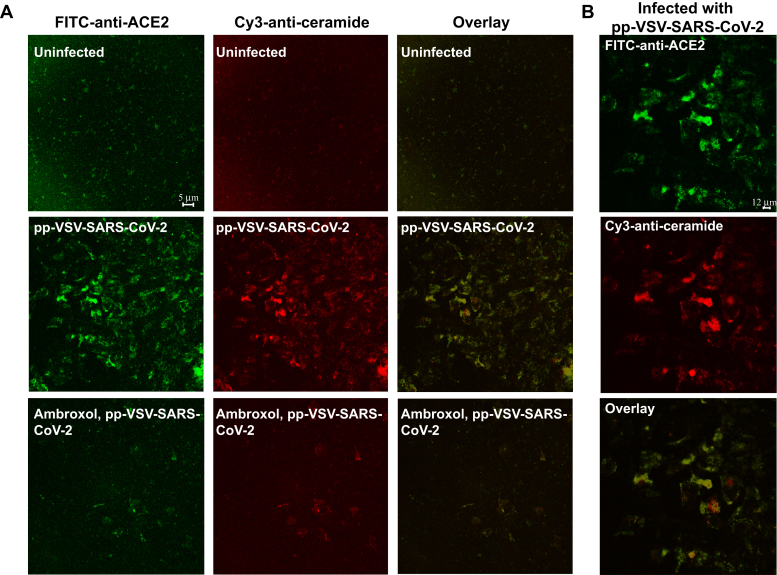
**ACE2 clusters in ceramide-enriched membrane domains upon infection.***A* and *B*, infection of Vero-E6 cells with pp-VSV-SARS-CoV-2 results in formation of ceramide-enriched membrane domains that cluster ACE2, which is prevented by treatment with ambroxol. Cells were infected for 30 min, fixed, stained with Cy3-coupled anti-ceramide and FITC–anti-ACE2 antibodies, and analyzed by confocal microscopy. *A*, shows a low magnification overview to visualize the abundance of ceramide-enriched membrane domains clustering ACE2. *B*, displays a higher magnification of infected cells. Please note that infection does not only induces ceramide but also increases surface expression of ACE2 (14). Shown is a representative result from six independent experiments. ACE2, angiotensin-converting enzyme 2; pp-VSV-SARS-CoV-2, vesicular stomatitis virus pseudoviral particles presenting SARS-CoV-2 spike protein on their surface.

We have previously shown that ceramide molecules form large ceramide-enriched membrane domains that serve to trap and cluster receptor molecules, thereby facilitating signaling *via* these receptors (10). Here, we demonstrate that infection of Vero-E6 cells with pp-VSV-SARS-CoV-2 spike results in the formation of ceramide-enriched membrane domains that cluster ACE2 (Fig. 3, *A* and *B*), suggesting that they serve as platforms to allow infection of the cells.

We have previously shown that sphingosine also inhibits infection of human cells with SARS-CoV-2 (23). We therefore tested whether ambroxol alters cellular sphingosine concentrations. The results demonstrate that even high concentrations of ambroxol did not change cellular sphingosine (not shown).

**Figure 4:**
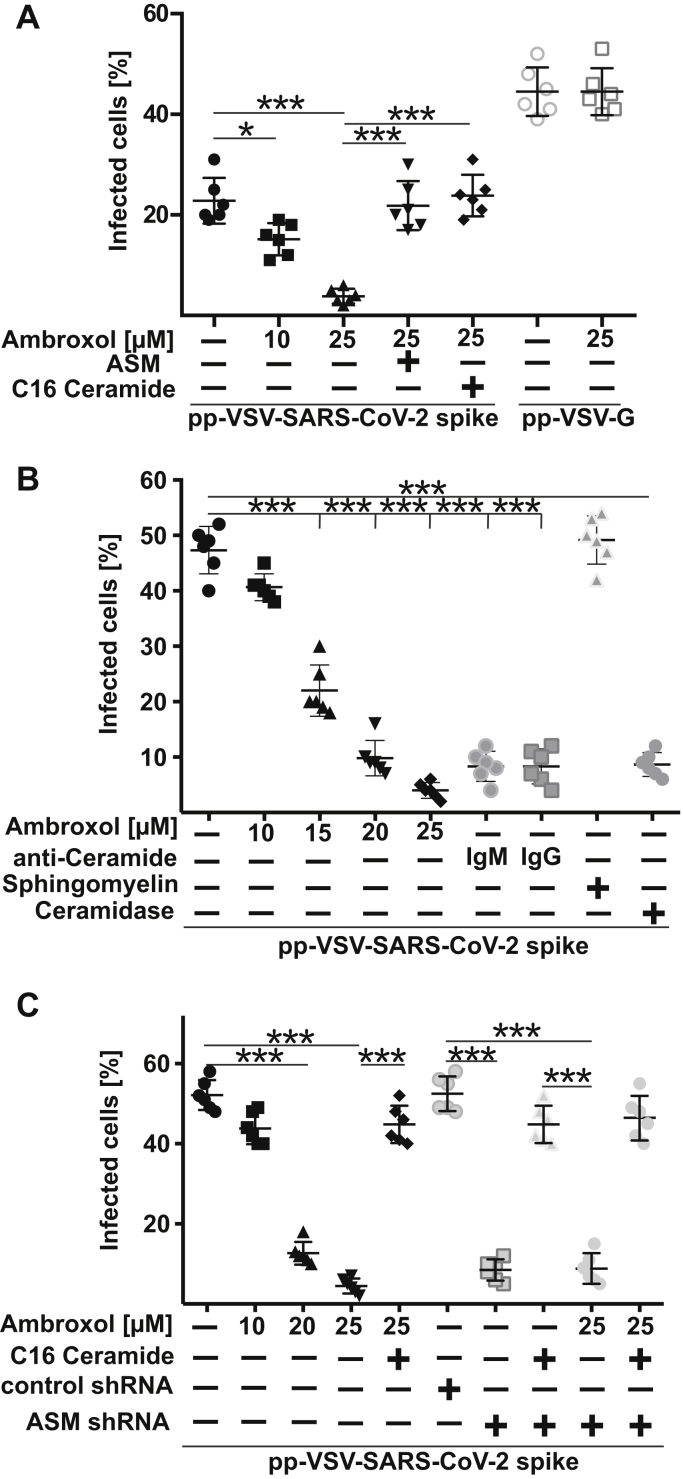
**Ambroxol prevents infection with SARS-CoV-2.***A*, Vero-E6 cells were pretreated with 10 μM or 25 μM ambroxol for 1 h or left untreated and were infected with pp-VSV-SARS-CoV-2 spike or pp-VSV-G for 24 h. Infection was measured by the expression of eGFP in the cells. Ambroxol blocked the infection of the cells with pp-VSV-SARS-CoV-2 spike (*closed symbols*) but had no influence on infection with pp-VSV-G (*open symbols*). Reconstitution of ceramide in Vero-E6 cells that had been treated with 25 μM ambroxol by the addition of 10 μM C16 ceramide or 0.2 U/ml acid sphingomyelinase (ASM) restored infection of the cells with pp-VSV-SARS-CoV-2 spike. Shown are the means ± SD of the percentage of infected cells from six independent experiments. ∗∗∗*p*< 0.001, ANOVA, followed by post hoc Student's *t* tests. *B*, higher concentrations of the pp-VSV-SARS-CoV-2 spike result in a higher percentage of infected cells but do not alter the ability of ambroxol to dose dependently inhibit infection of Vero-E6 cells. Neutralization of surface ceramide with two different anti-ceramide antibodies, that is, a monoclonal IgM antibody and an IgG antibody, or treatment with a ceramidase also prevented infection with pp-VSV-SARS-CoV-2 spike. Addition of sphingomyelin (50 μM) was without effect on the infection. Given are the means ± SD of the percentage of infected cells from six independent experiments. ∗∗∗*p*< 0.001, ANOVA, followed by post hoc Student's *t* tests. *Black symbols* are untreated and ambroxol-treated cells, and *gray symbols* represent cells constituted with anti-ceramide antibodies or sphingomyelin. *C*, treatment of Caco-2 cells with ambroxol dose dependently prevented infection with pp-VSV-SARS-CoV-2 spike. Likewise, transfection of Caco-2 cells with shRNA targeting the acid sphingomyelinase prevented infection, whereas a control shRNA had no effect on the infection. Addition of ambroxol to cells transfected with acid sphingomyelinase targeting shRNA had no additional effects. Displayed are the means ± SD of the percentage of infected cells from six independent experiments. ∗∗∗*p*< 0.001, ANOVA, followed by post hoc Student's *t* tests. *Closed symbols* are untransfected cells, and *open symbols* represent transfected cells. eGFP, enhanced GFP; pp-VSV-SARS-CoV-2, vesicular stomatitis virus pseudoviral particles presenting SARS-CoV-2 spike protein on their surface; SARS-CoV-2, severe acute respiratory syndrome coronavirus 2.

The blockade of pp-VSV-SARS-CoV-2 spike–dependent activation of acid sphingomyelinase activation and ceramide release by ambroxol promoted us to investigate whether ambroxol also inhibits entry of pp-VSV-SARS-CoV-2 spike. Vero-E6 cells were pretreated with ambroxol for 60 min, washed, and then infected with pp-VSV-SARS-CoV-2 spike for 60 min. The results indicate that 10 μM or 15 to 20 μM ambroxol reduced viral entry by approximately 50% or 80 to 90%, respectively, and 25 μM ambroxol completely blocked entry (Fig. 4, *A* and *B*). Infection with higher concentrations of VSV-SARS-CoV-2 spike was also blocked by ambroxol (Fig. 4*B*). Addition of purified acid sphingomyelinase that generates endogenous ceramide or 10 μM exogenous C16 ceramide restored viral entry (Fig. 4*A*) indicating that ambroxol acted *via* inhibition of the acid sphingomyelinase and the release of ceramide. In contrast, addition of sphingomyelin did not alter infection with pp-SARS-CoV-2 (Fig. 4*B*). Next, we neutralized ceramide with two different anti-ceramide antibodies or by incubation with ceramidase and show that these treatments also prevent viral uptake (Fig. 4*B*).

Treatment of Caco-2 cells with ambroxol or genetic downregulation of the acid sphingomyelinase in Caco-2 cells also resulted in an inhibition of pp-VSV-SARS-CoV-2 uptake, which was restored by C16 ceramide (Fig. 4*C*). Addition of ambroxol to C16 ceramide–treated acid sphingomyelinase downregulated cells did not further affect viral uptake supporting the notion that ambroxol specifically acts by targeting the acid sphingomyelinase.

Controls with pp-VSV-G, which does not activate the acid sphingomyelinase/ceramide system and does not employ this system for cellular infection as previously shown (14), demonstrated that pp-VSV-G entry was not modulated by ambroxol (Fig. 4*A*), indicating that ambroxol specifically blocks entry of pp-VSV-SARS-CoV-2 spike by inhibition of the acid sphingomyelinase/ceramide system.

**Figure 5:**
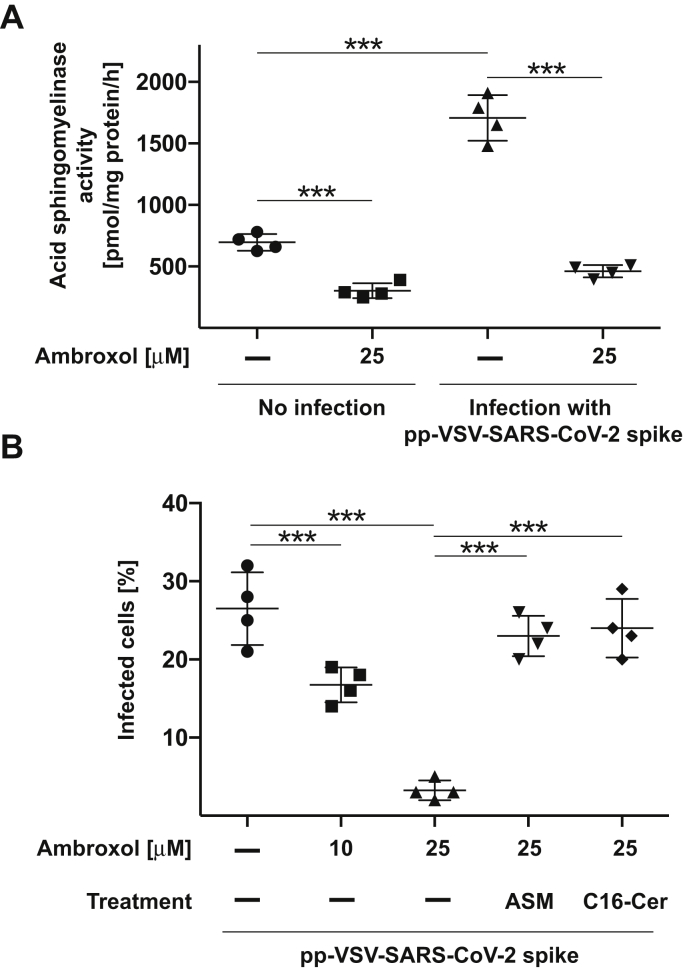
**Ambroxol prevents activation of the acid sphingomyelinase by an infection of freshly isolated human nasal epithelial cells with pp-VSV-SARS-Cov-2 spike *in vitro*.***A*, freshly isolated human nasal epithelial cells were incubated with 25 μM ambroxol for 1 h or left untreated and infected with pseudoviral particles of pp-VSV-SARS-CoV-2 spike for 30 min or left uninfected. The medium was removed, cells were lysed, and activity of the acid sphingomyelinase was determined by consumption of added [^14^C]sphingomyelin. Given are the means ± SD of the acid sphingomyelinase activity from four independent experiments. ∗∗∗*p* < 0.001, ANOVA, followed by post hoc Student's *t* tests. *B*, freshly isolated human nasal epithelial cells were incubated with 10 μM or 25 μM ambroxol for 1 h and infected with pp-VSV-SARS-CoV-2 spike. Infection was determined by counting eGFP-positive cells in at least 500 cells/sample. Reconstitution of ceramide by application of 0.2 U/ml purified acid sphingomyelinase, or 10 μM C16 ceramide (C16-Cer) restored infection in human nasal epithelial cells treated with ambroxol. Given are the means ± SD of the percentage of eGFP-positive, that is, infected cells, from four independent experiments. ∗∗∗*p* < 0.001, ANOVA followed by post hoc Student's *t* tests. eGFP, enhanced GFP; pp-VSV-SARS-Cov-2, vesicular stomatitis virus pseudoviral particles presenting SARS-CoV-2 spike protein on their surface.

We next examined whether our findings made with a cell line are recapitulated with primary human respiratory epithelial cells. To this end, we isolated nasal epithelial cells from volunteers, treated them with 25 μM ambroxol for 60 min, or left the cells untreated and determined the activity of the acid sphingomyelinase and infection of the cells. Ambroxol reduced the activity of the acid sphingomyelinase in uninfected cells by approximately 50% and prevented the stimulation of the acid sphingomyelinase upon exposure to pp-VSV-SARS-CoV-2 (Fig. 5*A*). Ambroxol blocked the entry of pp-VSV-SARS-CoV-2 spike into freshly isolated nasal epithelial cells (Fig. 5*B*) and addition of purified acid sphingomyelinase, or 10 μM C16 ceramide restored entry of the viral particles into ambroxol-treated cells (Fig. 5*B*).

**Figure 6:**
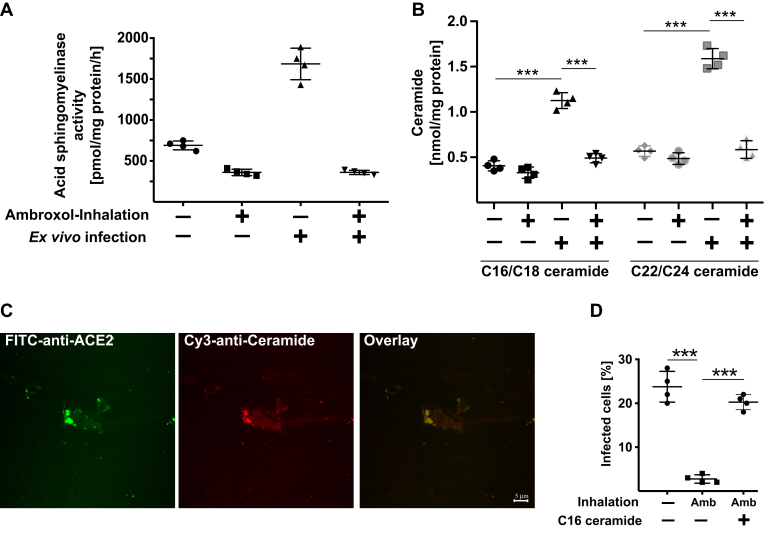
***In vivo* administration of ambroxol prevents infection of nasal epithelial cells with pp-VSV-SARS-CoV-2 spike *ex vivo.*** Nasal epithelial cells were obtained from volunteers. The volunteers then inhaled 2 ml ambroxol, that is, 15 mg ambroxol. Nasal epithelial cells were obtained from the opposite nasal cavity 1 h after inhalation. All cells were immediately washed after isolation, and the cell pellets were resuspended in cell culture medium and infected with pp-VSV-SARS-CoV-2 spike or left uninfected for 30 min (*A*–*C*) to determine acid sphingomyelinase (*A*), ceramide (*B*), and ceramide-enriched membrane domains with ACE2 clusters (*C*) or for 1 h (*D*) to determine infection (*D*). *A*, to determine acid sphingomyelinase activity, cell pellets were lysed in 50 mM sodium acetate (pH 5.0) and 0.2% NP-40, and the activity of the acid sphingomyelinase was determined by measuring the consumption of added [^14^C]sphingomyelin. *B*, cells were organically extracted, and ceramide was determined by the ceramide kinase assay method. *Black symbols* indicate C16/C18 ceramides, and *gray symbols* indicate C22/C24 ceramides. *C*, freshly isolated nasal epithelial cells were infected with pp-VSV-SARS-CoV-2 for 30 min, washed, fixed in 1% PFA for 10 min, washed and stained with Cy3-coupled anti-ceramide and FITC–anti-ACE2 antibodies. Shown are representative results from four independent experiments. *D*, cells were infected with pp-VSV-SARS-CoV-2 spike for 1 h, washed, and expression of eGFP was determined after 24 h in at least 500 epithelial cells per sample in randomly chosen microscopic fields. If indicated, C16 ceramide (10 μM) was reconstituted during the infection. *A*, *B*, and *D* show the means ± SD from four volunteers. ∗∗∗*p* < 0.001, ANOVA, followed by post hoc Student's *t* tests. ACE2, angiotensin-converting enzyme 2; Amb, ambroxol inhalation; eGFP, enhanced GFP; NP-40, Nonidet P40; PFA, paraformaldehyde; pp-VSV-SARS-CoV-2.

To simulate the effects of ambroxol on infection as closely as possible to treatment of patients, we isolated nasal epithelial cells from volunteers. The volunteers then inhaled 2 ml ambroxol (7.5 mg/ml), and nasal epithelial cells were again isolated from the opposite nasal cavity 60 min after inhalation and inoculated with pp-VSV-SARS-CoV-2 spike *ex vivo* for 60 min. Cells were washed, and infectious entry was determined after incubation for 24 h. In addition, we determined the activity of the acid sphingomyelinase in nasal epithelial cells prior and after inhalation of ambroxol. These studies revealed that inhalation of ambroxol *in vivo* markedly reduced the activity of the acid sphingomyelinase in nasal epithelial cells (Fig. 6*A*). Infection with pp-VSV-SARS-CoV-2 for 30 min resulted in an activation of the acid sphingomyelinase (Fig. 6*A*) and a release of ceramide (Fig. 6*B*), events that were blocked by a previous ambroxol inhalation. Infection also resulted in clustering of ACE2 in ceramide-enriched membrane domains of nasal epithelial cells (Fig. 6*C*). Most importantly, ambroxol inhalation prevented the *ex vivo* entry of pp-VSV-SARS-CoV-2 spike into freshly isolated nasal epithelial cells (Fig. 6*D*). Addition of C16 ceramide or recombinant acid sphingomyelinase restored infection of cells isolated from individuals that inhaled ambroxol, with pp-VSV-SARS-CoV-2 spike (Fig. 6*D*).

## Discussion

**Figure 7:**
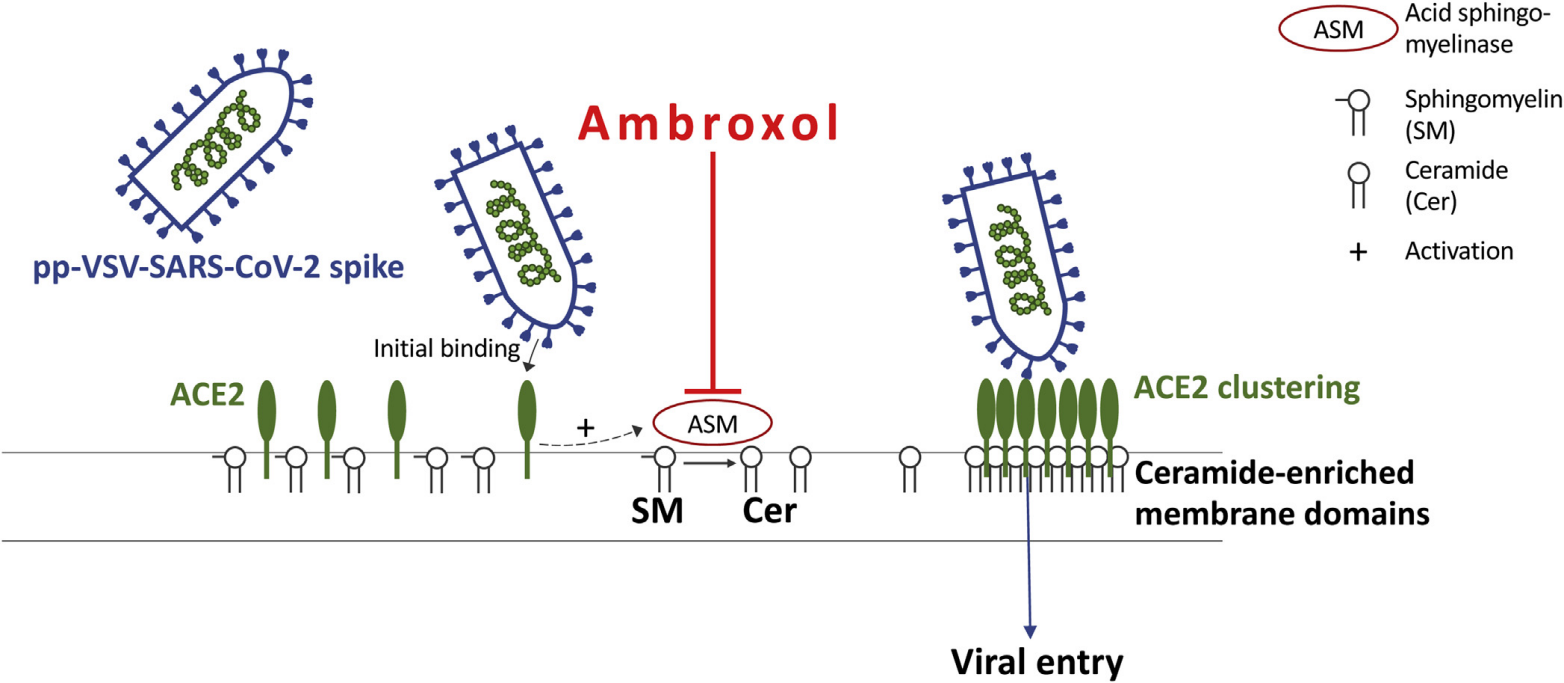
**Scheme of the proposed inhibition of pp-SARS-CoV-2 spike infections by ambroxol.** Initial binding of pp-VSV-SARS-CoV-2 spike results in activation of the acid sphingomyelinase, formation of surface ceramide, and formation of ceramide-enriched membrane platforms that serve to trap and cluster activated ACE2 receptors. The clustering of ACE2 in ceramide-enriched membrane platforms might facilitate signaling *via* this receptor finally resulting in internalization but also brings the virus into close proximity to proteases required for uptake. ACE2, angiotensin-converting enzyme 2; pp-VSV-SARS-CoV-2, vesicular stomatitis virus pseudoviral particles presenting SARS-CoV-2 spike protein on their surface.

The present studies demonstrate that ambroxol prevents the entry of pp-VSV-SARS-CoV-2 spike into cultured epithelial cells as well as freshly isolated human nasal epithelial cells *ex vivo* (Fig. 7). Most importantly, inhalation of ambroxol was sufficient to reduce the activity of the acid sphingomyelinase in nasal epithelial cells *in vivo* and prevent an infection with pp-VSV-SARS-CoV-2 spike of nasal epithelial cells *ex vivo*. The inhalation experiments used standard concentrations of ambroxol that are also used for treatment of patients. These concentrations were sufficient to reduce acid sphingomyelinase activity in nasal epithelial cells *in vivo*, as shown here. Thus, clinically used concentrations should be sufficient to prevent infection with pp-VSV-SARS-CoV-2 spike. We inhaled an approximately 20 mM solution of ambroxol. Since 20 to 25 μM ambroxol is sufficient to block infection with pp-VSV-SARS-CoV-2, we expect a very broad therapeutic window.

We and others have shown that ceramide often promotes bacterial and viral infections (24, 25, 26, 27). Ceramide alters the plasma membrane and induces the formation of large ceramide-enriched membrane platforms that serve to trap and cluster receptor molecules and their associated signalosome (7). Here, we provide evidence that ACE2 clusters in ceramide-enriched membrane domains upon inoculation of cells with pp-VSV-SARS-CoV-2 spike supporting the idea that ACE2 needs to be reorganized into these domains to mediate infection. It is also possible that ceramide-enriched membrane platforms are required for activation of transmembrane serine protease 2 (TMPRSS2) and/or cathepsin L that mediate processing of spike required for fusion with the cell membrane (28, 29).

We assume that uptake of the viral particles occurs 20 to 40 min after infection in a relatively coordinated manner and that this results in a relatively short-lived activation of the acid sphingomyelinase. We also assume that ceramide is rapidly consumed. It will be very interesting to define whether ceramide is converted to a glycosylated product, phosphorylated, converted to sphingosine, sphingosine 1-phosphate, and hexadecenal, or even used to produce sphingomyelin again. It will be also very interesting to perform detailed lipidomics upon SARS-CoV-2 infection.

A role of the acid sphingomyelinase/ceramide system for viral infections of cells has been previously shown for rhinoviruses, Ebola, measles, and Japanese encephalitis virus (25, 30, 31, 32). Treatment with FIASMA prevented cellular entry of these viruses (25, 30, 31, 32) indicating the significance of the acid sphingomyelinase/ceramide system for viral infection. In contrast to the proinfective function of ceramide described previously, it was shown that infection with influenza, which also results in ceramide release, although by the *de novo* pathway, is inhibited by ceramide (33). Ceramide prevented replication of influenza virus, suggesting that ceramide plays a protective and antiviral role. Further studies demonstrated that genetic deficiency or pharmacological inhibition of the acid sphingomyelinase did not affect cellular infection with influenza virus (34), suggesting that different pools of ceramide have different functions in viral infections.

In contrast to ceramide that promotes viral infection with SARS-CoV-2, sphingosine (2-amino-4-trans-octadecene-1,3-diol), which is released from ceramide by the activity of neutral, acid, or alkaline ceramidases, was recently shown to exert antiviral activity, either by trapping viruses in endosomes and thereby shuttling them to lysosomal degradation (35) or by competing the binding of SARS-CoV-2 spike to ACE2 (23).

Host cell entry of SARS-CoV-2 is initiated by the binding of the surface unit S1 of the viral spike glycoprotein to its cellular receptor ACE2, resulting in cleavage of the viral spike protein by the activity of TMPRSS2 or the endosomal cysteine protease cathepsin L (22, 28, 29).

Bromhexine, which is structurally very similar to ambroxol, has been shown to inhibit TMPRSS2 (36), the activity of which is essential for SARS-CoV-2 infection of lung cells. However, in our studies, inhibition of the uptake was restored by addition of purified acid sphingomyelinase or exogenous C16 ceramide, suggesting that ambroxol primarily regulates viral entry *via* the acid sphingomyelinase/ceramide system.

Functional inhibitors of the acid sphingomyelinase are usually cationic amphiphilic drugs that diffuse into lysosomes because of their physicochemical properties, that is, high lipophilicity and weak basicity (15, 16, 17, 18, 19, 20, 21). The drugs are then protonated and thereby trapped in lysosomes. The lipophilic and organic ring system of FIASMAs may bind to the lipid membrane, whereas the protonated tertiary amine displaces acid sphingomyelinase from lysosomal membranes or from the plasma membrane within acidic subdomains (15, 16, 17, 18, 19, 20, 21). The displacement of the enzyme from lysosomal membranes, in particular intralysosomal vesicles, releases the enzyme into the lysosomal lumen where it is degraded (15, 16, 17, 18, 19, 20, 21). Thus, these drugs indirectly inhibit acid sphingomyelinase activity.

Our data indicate a central role of the acid sphingomyelinase and ceramide for infection of human cells with pp-SARS-CoV-2 spike. Thus, any treatment that increases the concentration of ceramide, in particular surface ceramide, might result in increased infection with SARS-CoV-2. Ambroxol did not influence acid ceramidase activity at concentrations that inhibited the acid sphingomyelinase supporting the notion that the drug specifically acts by blocking the acid sphingomyelinase. However, downregulation of the acid ceramidase, for instance by bacterial infections (37), might result in increased ceramide levels and, thus, might promote infection with SARS-CoV-2.

Infection with mutants of SARS-CoV-2 that might escape the immune system and are presently of great interest would be still inhibited with ambroxol treatment, since all these mutants require ACE2 for infection. Activation of the acid sphingomyelinase and release of ceramide are downstream events of the interaction of SARS-CoV-2. Thus, targeting the acid sphingomyelinase/ceramide system might be a very interesting approach to prevent infection with SARS-CoV-2 mutants.

Our study is supported by two prepublished studies. A study by Bradfute *et al*. (38) demonstrated that ambroxol blocks infection of Vero-E6 cells with SARS-CoV-2. A study by Olaleye *et al*. (39) revealed that micromolar concentrations of ambroxol prevented cellular infections with SARS-CoV-2 determined as cytopathic effect of the virus. The authors further demonstrated using recombinant proteins of the receptor-binding domain of spike and ACE2 that ambroxol interferes with the binding of SARS-CoV-2 spike with human ACE2 *in vitro* (39). Thus, ambroxol may prevent infections with SARS-CoV-2 at different levels, that is, blocking the binding of the viral spike protein to its human receptor, and by inhibition of the acid sphingomyelinase/ceramide system.

Here, we detected that inhalation of ambroxol was sufficient to reduce acid sphingomyelinase in nasal epithelial cells *in vivo* and to block pp-VSV-SARS-CoV-2 spike-driven entry into these cells *ex vivo*. This suggests that inhalation of ambroxol may prevent SARS-CoV-2 infection and spread in the human respiratory epithelium.

We have previously shown that antidepressants such as fluoxetine and amitriptyline and several other antidepressants prevent the infection of human cells with SARS-COV-2 (14). These antidepressants also inhibit the acid sphingomyelinase and act very similar to ambroxol. However, antidepressants must be given systemically, while ambroxol can be inhaled and only acts in the respiratory tract. Ambroxol is very safe and well tolerated and can in principle be applied with no temporal limitation. Antidepressants have a variety of side effects (*e.g.*, long-QT syndrome), drug interactions (*e.g.*, inhibition of CYP2D6), and contraindications (*e.g.*, parallel use of monoamine oxidase inhibitors) must be considered, none of which are important with ambroxol. The particularly good tolerability of ambroxol could justify the low-threshold use of ambroxol, for example, prophylactically in persons at risk to develop severe COVID-19 infections, such as elderly individuals, or after contact with an infected individual or after testing positive for SARS-CoV-2 without symptoms of COVID-19. However, ambroxol is very likely not effective at later stages when the viral infection becomes systemic.

Ambroxol is a safe drug that has been used clinically since 1979, at least in Germany. It has almost no side effects. In addition, inhalation is a very safe method of treatment in general very often avoiding systemic side effects. Thus, our data justify further studies to investigate whether ambroxol can be clinically used to prevent or treat infections with SARS-CoV-2.

## Experimental procedures

### Ethics statement

The experiments were approved by the ethics committee of the University Hospital Essen under the numbers 19-9033-BO and 276235-BO. The experiments are in accordance with the Declaration of Helsinki principles.

### Human studies

Human nasal epithelial cells were obtained from four healthy volunteers, one female and three males (aged 48, 54, 54, and 56). We have already shown that infection with pp-SARS-CoV-2 spike does not differ between cells from women or men. Sample size was planned for the continuous variable difference in viral uptake and was based on two-sided Wilcoxon–Mann–Whitney tests using G∗Power, version 3.1.7 (University of Duesseldorf, Germany). Nasal epithelial cells were obtained from all subjects prior and after inhalation of ambroxol and, thus, all subjects were included in both arms (untreated *versus* treated) of the study. Subjects inhaled a total amount of 15 mg ambroxol (7.5 mg/ml) by using a Pariboy R. Ambroxol was from Ratiopharm.

Nasal epithelial cells from these volunteers were obtained immediately before and 1 h after ambroxol inhalation. The cells were removed from the nasal mucosa by inserting a small brush into the nose (approximately 1.5–2 cm deep). The brush was gently rotated within the nose. Nasal epithelial cells were released from the brush and suspended immediately in 1 ml HEPES/saline (H/S; 132 mM NaCl, 20 mM HEPES [pH 7.4], 5 mM KCl, 1 mM CaCl_2_, 0.7 mM MgCl_2_, and 0.8 mM MgSO_4_), pelleted, and immediately used for infection.

### Pseudoviral particles

Pseudotyped viral particles were generated according to a previously published protocol (40). These particles are based on a replication-deficient VSV that codes for enhanced GFP (eGFP) and firefly luciferase instead of parental VSV-G (VSV∗ΔG-FLuc) (41). Human embryonic kidney 293T cells were transfected with the appropriate gene constructs for 24 h using the calcium-phosphate method to transiently express either SARS-CoV-2 spike or VSV-G. Cells were inoculated with VSV-G-transcomplemented VSV∗ΔG-FLuc for 1 h at 37 °C and 5% CO_2_, the inoculum was removed, cells were washed with PBS, fresh culture medium was added, and cells were further incubated at 37 °C and 5% CO_2_. In cells expressing SARS-CoV-2 spike, the culture medium was supplemented with anti-VSV-G antibody (I1, mouse hybridoma supernatant from American Type Culture Collection [ATCC] CRL-2700) to achieve inactivation of residual VSV-G-transcomplemented VSV∗ΔG-FLuc. All culture supernatants were harvested 16 h after inoculation, cellular debris was pelleted by centrifugation (4000*g*, 4 °C, 10 min), and the supernatants were used for the experiments. Pseudoviral particles were concentrated using a spin column to remove medium.

### Vero-E6 and Caco-2 cells

Vero-E6 cells (ATCC CCL-81; monkey kidney epithelial cells) were grown in Dulbecco's modified Eagle's medium (DMEM) supplemented with 10 mM Hepes (pH 7.4; Carl Roth GmbH), 2 mM l-glutamine, 1 mM sodium pyruvate, 100 μM nonessential amino acids, 100 U/ml penicillin, 100 μg/ml streptomycin, and 10% fetal calf serum (FCS).

Caco-2 colon epithelial cells (ATCC HTB-37) were grown in minimum essential medium (MEM) supplemented as aforementioned.

### Cellular infection

#### Cell lines

Vero-E6 and Caco-2 cells were grown to subconfluency for 24 h on glass coverslips in a 24-well plate for 24 h, washed and treated with 1, 2.5, 5, 10, 25, 50, or 75 μM ambroxol for 1 h prior to infection with pp-VSV-SARS-CoV-2 spike or pp-VSV-G particles. Ambroxol (stock 7.5 mg/ml) was diluted in H/S and then directly added to the cells. Control (uninfected) samples were treated with the solvent of ambroxol. The medium was then removed, 250 μl of a cell culture supernatant containing pp-VSV-SARS-CoV-2 or pp-VSV-G particles and, if indicated, ambroxol at the previous concentration was added, the cells were incubated for 24 h, washed once in H/S, fixed in 1% paraformaldehyde (PFA) buffered with PBS (pH 7.3) for 10 min, washed, embedded in Mowiol, and analyzed with a Leica TCS-SL confocal microscope (Leica Microsystems) equipped with a 40× lens and Leica LCS software, version 2.61 (Leica Microsystems). We counted eGFP-positive cells in at least 500 cells per sample in randomly chosen microscopic fields.

#### Nasal epithelial cells

Nasal epithelial cells were obtained from untreated volunteers or volunteers who had inhaled ambroxol, resuspended in H/S, aliquoted, and pelleted by centrifugation at 2800 rpm for 8 min in an Eppendorf centrifuge (1710*g*). Cells were then resuspended in MEM supplemented with 10 mM HEPES (pH 7.4; Carl Roth GmbH), 2 mM l-glutamine, 1 mM sodium pyruvate, 100 μM nonessential amino acids, 100 U/ml penicillin, 100 μg/ml streptomycin, and 10% FCS, treated with ambroxol for 1 h or left untreated, pelleted, and infected with pp-VSV-SARS-CoV-2 spike particles for 60 min. Cells from patients prior or after inhalation of ambroxol were immediately infected with pp-SARS-CoV-2 spike for 1 h. Cells were then washed and further incubated for 24 h to allow expression of eGFP. Infection was analyzed as aforementioned in at least 500 epithelial cells per sample.

Aliquots of the nasal epithelial cells were immediately shock frozen in liquid nitrogen after removal for determination of acid sphingomyelinase activity or ceramide concentrations (see later).

### FITC–annexin V binding

Vero cells were incubated for 24 h with 25 μM ambroxol in MEM supplemented as previously, washed, trypsinized, washed in H/S, and stained for 15 min with FITC–annexin V according to the instructions of the vendor, and analyzed by flow cytometry with a FACS Calibur (Becton Dickinson). Controls were permeabilized for 5 min with 0.1% Triton X-100 at room temperature before incubation with FITC–annexin V.

### Acid sphingomyelinase activity

Vero-E6 or nasal epithelial cells were left untreated or pretreated with ambroxol for 1 h, washed once in H/S, and the pellets were immediately shock frozen or resuspended and infected with 250 μl of cell culture supernatant containing pp-VSV-SARS-CoV-2 spike for 10, 20, 30, or 45 min. Untreated control cells were incubated with DMEM or MEM that was supplemented as aforementioned. Supernatants were removed, and the samples were shock frozen, thawed, and immediately lysed in 250 mM sodium acetate (pH 5.0) and 1% Nonidet P40 (NP-40) 5 min. Lysates were diluted to 0.2% NP-40 in 250 mM sodium acetate (pH 5.0), and 0.05 μCi [^14^C]sphingomyelin (52 mCi/mmol; PerkinElmer, #NEC 663010UC) per sample was added. The substrate [^14^C]sphingomyelin was dried for 10 min in a SpeedVac, resuspended in 250 mM sodium acetate (pH 5.0) and 0.1% NP-40, and sonicated for 10 min in a bath sonicator before it was added to the samples. The samples were incubated for 30 min at 37 °C with shaking at 300 rpm. Samples were then organically extracted in four volumes of CHCl_3_:CH_3_OH (2:1, v/v), vortexed, the samples were centrifuged, and an aliquot of the upper aqueous phase was scintillation counted to determine the release of [^14^C]phosphorylcholine from [^14^C]sphingomyelin.

### Measurement of ceramide

Cells were treated with ambroxol for 1 h or left untreated as aforementioned, the medium was removed, cells were lysed in 200 μl H_2_O, and the lysates were extracted in CHCl_3_:CH_3_OH:1 N HCl (100:100:1, v/v/v). Phases were separated by 5 min at 14,000 rpm centrifugation of the samples. The lower phase was collected, dried, and resuspended in 20 μl of a detergent solution (7.5% [w/v] *n*-octyl-d-glucopyranoside (OGP), 5 mM cardiolipin in 1 mM diethylenetriaminepentaacetic acid [DTPA]). Micelles were obtained by bath sonication for 10 min and used for the kinase reaction, which was initiated by adding 70 μl of a reaction mixture containing 10 μl diacylglycerol kinase (GE Healthcare Europe), 0.1 M imidazole/HCl (pH 6.6), 0.2 mM DTPA (pH 6.6), 70 mM NaCl, 17 mM MgCl_2_, 1.4 mM ethylene glycol tetraacetic acid, 2 mM dithiothreitol, 1 μM ATP, and 5 μCi [^32^P]γATP (6000 Ci/mmol; Hartmann Radiochemicals). The kinase reaction was performed for 60 min at room temperature with 300 rpm shaking. Samples were then organically extracted in 1 ml CHCl_3_:CH_3_OH:1 N HCl (100:100:1, v/v/v), 170 μl buffered saline solution (135 mM NaCl, 1.5 mM CaCl_2_, 0.5 mM MgCl_2_, 5.6 mM glucose, and 10 mM Hepes [pH 7.2]), and 30 μl of a 100 mM EDTA solution was added, and the phases were separated. The lower phase was collected, dried in a SpeedVac, separated on Silica G60 TLC plates with chloroform/acetone/methanol/acetic acid/H_2_O (50:20:15:10:5, v/v/v/v/v), and developed with a Fujifilm FLA-3000 Fluorescence Laser Imaging Scanner (Fuji). Ceramide amounts were determined by comparison with a standard curve using C16 to C24 ceramides as substrates.

### Ceramide reconstitution

Cells were treated with 25 μM ambroxol for 1 h and infected with pp-VSV-SARS-CoV-2 spike in the presence or absence of 10 μM C16 ceramide (Avanti Polar Lipids; #860516) or 0.2 U/ml recombinant acid sphingomyelinase (specific activity: 1700 pmol/min/μg; R&D, #5348-PD-010). C16 ceramide was resuspended in cell culture medium and sonicated for 10 min in a bath sonicator before use. We then determined viral uptake as aforementioned.

### Sphingomyelin treatment

Sphingomyelin was suspended at 10 mM in 10% OGP, diluted in DMEM/10% FCS to 50 μM, sonicated for 10 min, and added to the cells for 60 min prior any infection. The sphingomyelin concentration was maintained at 50 μM during the infection. Sphingomyelin uptake was controlled by addition of 0.05 μCi [^14^C]sphingomyelin that was prepared as aforementioned. Cells were extensively washed after 60 min of incubation, and [^14^C]sphingomyelin uptake was determined by liquid scintillation counting of cell lysates.

### Genetic downregulation of acid sphingomyelinase

The expression of acid sphingomyelinase in Caco-2 cells was downregulated by transient transfection with commercial shRNA targeting acid sphingomyelinase (Santa Cruz Inc; #sc-41650) employing electroporation at 400 V with five pulses, 3 ms each, with a BTX electroporator. Controls were transfected with an irrelevant shRNA (Santa Cruz Inc, #sc-108060). Dead cells were removed after 24 h, and cells were cultured for an additional 24 h prior to infection with pp-VSV-SARS-CoV-2 spike. Downregulation of acid sphingomyelinase was confirmed by measuring the activity of the enzyme in transfected, control-transfected, and untransfected cells.

### Confocal microscopy of ceramide and ACE2

Vero-E6 cells were cultured on glass cover slips overnight, treated with 25 μM ambroxol for 1 h as aforementioned or left untreated, and infected with pp-VSV-SARS-CoV-2 spike or in the presence or absence of 25 μM ambroxol for 60 min or left uninfected. The cells were washed once in PBS (pH 7.4) and fixed for 10 min in 1% PFA buffered in PBS. Freshly isolated nasal epithelial cells were infected with pp-VSV-SARS-CoV-2 spike for 30 min, washed, and fixed for 10 min in 1% PFA buffered in PBS. The cells were then washed twice in PBS, blocked by incubation for 15 min in H/S + 5% FCS, washed once, and stained with anti-ceramide antibodies (1:200; clone S58-9) in H/S with 1% FCS for 45 min at room temperature. Cells were washed three times in PBS plus 0.05% Tween-20. The samples were then incubated with goat anti-ACE2 antibodies (1:500; R&D; #AF 933) for 45 min at room temperature. Samples were washed again and consecutively stained with FITC-anti-goat F(ab)_2_ fragments and Cy3-anti-mouse IgM F(ab)_2_ fragments. Samples were washed between and after the stainings and finally embedded in Mowiol. Control stainings were performed with irrelevant IgM and goat IgG and showed no positive staining. We also included controls with secondary Cy3- or FITC-coupled antibodies only.

Samples were analyzed by confocal microscopy with a Leica TCS-SP5 confocal microscope equipped with a 40× lens and Leica LCS software, version 2.61. All samples of the same series were measured at identical settings.

### Ceramide neutralization

Ceramide was neutralized in Vero-E6 cells by addition of 50 μg/ml of the anticeramide IgM antibody clone S85-9 (Glycobiotech; MAB_0014) or of 100 μg/ml of a mouse monoclonal anticeramide IgG (Antibody Research Corp; #111583), or 0.2 units/ml of neutral ceramidase (R&D; #3557 AH) to the cells. Cells were incubated for 10 min and then infected with pp-VSV-SARS-CoV-2 spike particles for 60 min. Controls were incubated with irrelevant IgM or IgG antibodies (Dako). Cells were then washed, cultured for 24 h, and analyzed for infection as aforementioned.

### Acid ceramidase activity

Vero-E6 cells were treated with 25 μM ambroxol or left untreated and lysed in 1% NP-40 in 150 mM sodium acetate (pH 4.5) for 5 min on ice, diluted to 0.1% NP-40 in 150 mM sodium acetate (pH 4.5), and 0.3 μCi/sample [^14^C_16_]ceramide (ARC0831; 55 mCi/mmol) was added. The substrate [^14^C_16_]ceramide was dried for 10 min, resuspended in 0.1% OGP in 150 mM sodium acetate (pH 4.5), and bath sonicated for 10 min prior to addition to the samples. The samples were incubated at 37 °C for 60 min. The reaction was terminated by extraction in H_2_O and CHCl_3_:CH_3_OH:HCl (100:100:1, v/v/v). The lower phase was dried, and samples were resuspended in CHCl_3_:CH_3_OH (1:1, v/v), separated by TLC with CHCl_3_:CH_3_OH:NH_4_OH (90:20:0.5, v/v/v), and analyzed with a Fujifilm FLA-3000 Flourescence Laser Imaging Scanner (Fuji).

### Sphingosine measurements

Vero-E6 cells were treated with 25 μM ambroxol for 60 min, the medium was removed, cells were scrapped from the plate in 200 μl H_2_O, and extracted in 800 μl CHCl_3_:CH_3_OH:1 N HCl (100:200:1, v/v/v). The lower phase was dried and resuspended in a detergent solution (7.5% [w/v] OGP and 5 mM cardiolipin in 1 mM DTPA). The kinase reaction was performed by addition of 0.001 units of sphingosine kinase in 50 mM Hepes (pH 7.4), 250 mM NaCl, 30 mM MgCl_2_, 1 mM ATP, and 10 μCi [^32^P]γATP. Samples were incubated for 60 min at 37 °C with shaking (350 rpm). The reaction was terminated by adding 100 μl H_2_O, 20 μl 1 N HCl, 800 μl CHCl_3_:CH_3_OH:1 N HCl (100:200:1, v/v/v), and 240 μl each of CHCl_3_ and 2 M KCl. Samples were vortexed, phases were separated, the lower phase was collected, dried, dissolved in 20 μl CHCl_3_:CH_3_OH (1:1, v/v), and separated on Silica G60 TLC plates with CHCl_3_:CH_3_OH:acetic acid:H_2_O (90:90:15:5, v/v/v/v) as developing solvent. The TLC plates were analyzed with a phosphorimager. Sphingosine levels were determined with a standard curve of C18-sphingosine.

### Statistical analysis

Data are expressed as arithmetic means ± SD. For the comparison of continuous variables from independent groups, we used one-way ANOVA followed by post hoc Student's *t* tests for all pairwise comparisons and the Bonferroni correction for multiple testing. The *p* values for the pairwise comparisons were calculated after Bonferroni correction. All values were normally distributed. Statistical significance was set at the level of *p* ≤ 0.05 (two-tailed). Sample size planning for the continuous variable *in vivo* experiments was based on two-sided Wilcoxon–Mann–Whitney tests (free software: G∗Power, version 3.1.7). Investigators were blinded to the identity of the samples in all microscopy experiments.

## Data availability

All data are available upon request from the authors. Authors confirm that all data are included in the article.

## Conflict of interest

The authors declare that they have no conflicts of interest with the contents of this article.
